# More Light? Opportunities and Pitfalls in Digitalized Psychotherapy Process Research

**DOI:** 10.3389/fpsyg.2021.544129

**Published:** 2021-03-19

**Authors:** Matthias Domhardt, Pim Cuijpers, David Daniel Ebert, Harald Baumeister

**Affiliations:** ^1^Department of Clinical Psychology and Psychotherapy, Ulm University, Ulm, Germany; ^2^Department of Clinical, Neuro- and Developmental Psychology, Vrije Universiteit Amsterdam, Amsterdam, Netherlands

**Keywords:** psychotherapy, mechanisms of change, e- and m-health, mediators, components, active ingredients, digital factors

## Abstract

While the evidence on the effectiveness of different psychotherapies is often strong, it is not settled *whereby* and *how* these therapies work. Knowledge on the causal factors and change mechanisms is of high clinical and public relevance, as it contributes to the empirically informed advancement of psychotherapeutic interventions. Here, digitalized research approaches might possess the potential to generate new insights into human behavior change, contributing to augmented interventions and mental healthcare practices with better treatment outcomes. In this perspective article, we describe recent findings of research into change mechanisms that were only feasible with digital tools and outline important future directions for this rather novel branch of research. Furthermore, we indicate several challenges and pitfalls that are to be solved, in order to advance digitalized psychotherapy process research, both methodologically and technologically.

## Introduction

Despite decades of research efforts to unveil the working mechanisms in psychotherapies for common mental disorders, the evidence base on the causal factors and therapeutic processes in most of these interventions remains largely uncertain (Cuijpers et al., 2019). Most researchers would probably agree that comprehensive knowledge on the mechanisms of change (i.e., the actual processes responsible for change) is central to develop more powerful intervention packages with optimized outcomes though. We highlight that certain features of digitalization convey novel opportunities for psychotherapy process research, which hold the potential to lift this kind of research on another level and shed *more light* upon an enduring *black box*. At the same time, we also point to important challenges and hurdles that might obstruct the full evolvement of this new branch of research.

Here, we conceive *digitalized* psychotherapy process research rather broadly, comprising different methods and means, which share the commonality that they are all technologically realized and were not available to prior psychotherapy research of the pre-digital age. Established examples of these digital approaches are video-taped analyses of therapeutic processes (e.g., Koole and Tschacher, 2016), videoconference-based psychotherapy (e.g., Etzelmueller et al., 2018), or routine outcome monitoring (e.g., Lutz, 2002; Lambert et al., 2018). These digital tools might be predominantly applied for research purposes only, but might also directly support psychotherapeutic practices in clinical routine. Most prominently, eHealth and mHealth interventions (i.e., psychotherapeutic treatment programs that are either delivered via personal computers and web-browsers, or smartphones and mobile applications, respectively) are extensively researched in recent years and show promise to extend mental healthcare, given their particular features, like flexibility and anonymity in conduct, possible cost-effectiveness, and outreach on a population scale (Andersson et al., 2019; Linardon et al., 2019; Domhardt et al., 2020a). Moreover, internet- and mobile-based interventions (IMIs) might not only augment the capabilities in mental healthcare (Ebert et al., 2017) but also hold a considerable potential for psychotherapy research on change mechanisms because of their specific properties.

## Novel Methodological and Technological Opportunities

To begin with, a major asset of the implementation of IMIs in psychotherapy process research is the possibility to reach a higher standardization of interventions and their components, which was not possible with previous conventional research approaches within face-to-face therapy settings. This methodological progression enables a more reliable detection of the effects of single components in dismantling and additive design studies (Steubl et al., 2019), as previously hard to control confounds, like therapist factors (e.g., personal views, professional experience, and skills) or the actual presentation of manualized therapeutic content, can be hold constant. Thereby, dismantling studies have revealed several important insights so far, for example, the superiority of IMIs with guidance compared to pure self-help interventions (Baumeister et al., 2014) or the comparable effectiveness of transdiagnostic and disorder-specific interventions (Domhardt et al., 2019). These preliminary findings suggest that the therapeutic alliance might play a prominent role as common factor in digitalized psychotherapeutic interventions as well (Berger, 2016), and the potential of IMIs for scalability purposes might be further amplified by means of transdiagnostic treatment manuals (e.g., Weisel et al., 2019). Yet, future studies must expand our knowledge by disentangling the incremental or surrogating effects of central other components, like automation of support (as a possible cost-efficient alternative of human support in IMIs) and tailoring of intervention content to patients’ needs (in contrast to “one-size-fits-all”-interventions), in order to fully grasp the actual potential and limitations of IMIs to extend and augment mental healthcare efforts on a global scale.

Another advantage of experimental studies with IMIs is that they enable an unprecedented way to break down the utterly complex and dynamic processes of psychotherapeutic interventions into paradigmatic fragments, with the direct manipulation of isolated and clearly operationalized specific factors. In this sense, digital interventions might serve as a “mouse model” for psychotherapy process research and allow for the evaluation of distinct psychological and biological mechanisms of therapeutic change in original experimental designs. For instance, Hirsch et al. (2018) investigated the effects of experimentally inducing positive interpretations by means of a priming task before internet-delivered cognitive bias modification training (CBM) in patients with symptoms of depression and anxiety. The authors found that changes in interpretation bias partially mediated the effects of CBM on worry and rumination at follow-up, contributing to our understanding of the causal role of interpretation bias in worry and rumination, as a relevant target for face-to-face and online psychotherapy alike (Hirsch et al., 2018).

Moreover, ecological momentary assessment (EMA) and smart sensing studies make the step out of laboratories and facilitate the immediate detection of variables, irrespective of the constraints of space and time (Myin-Germeys et al., 2018). This can eventually lead to more valid multimodal assessments (i.e., “digital phenotyping”; Jain et al., 2015; Baumeister and Montag, 2019), free from several biases (e.g., recall, social desirability; Shiffman et al., 2008) and without overlapping measurements of outcome and mediator constructs in ordinary paper–pencil self-reports with similar—or even identical—items. Future research is needed however, to investigate, if novel biases arise within EMA studies themselves (e.g., reactive assessment; van Ballegooijen et al., 2016). The ease and high-intensity of data collection with EMA (Schuster et al., 2020) and digital tools will ultimately lead to larger sample sizes and big data sets that would alleviate the problem of limited statistical power, which is a long-lasting impediment of psychotherapy (process) research (Domhardt et al., 2021). This assumption is corroborated in a recent review, showing that mediation studies with IMIs for depression (Domhardt et al., 2021) exhibit a substantial larger amount of study participants on average (M = 262, SD = 243), when compared to conventional psychotherapy process research for depression (Lemmens et al., 2016; M = 173, SD = 145).

Fine-grained longitudinal data on therapeutic processes, gathered within or outside therapy sessions, can be shared among researchers conducting individual patient data meta-analyses, in order to develop multivariable algorithms that contribute to precision mental health (Furukawa et al., 2018, 2019; Lin et al., 2019). Innovative machine learning approaches might predict trajectories of change based on these data, which can inform pre-treatment and in-session decisions of mental healthcare practices (Cohen and DeRubeis, 2018; Goldberg et al., 2020; Rubel et al., 2020). Additionally, virtual reality (VR) interventions reveal novel findings on change mechanisms that were not conceivable with conventional studies so far. For instance, in their original study Pot-Kolder and colleagues randomized 116 patients with psychotic disorders either to VR-based Cognitive Behavior Therapy (CBT) or waitlist (treatment as usual). The VR-CBT intervention consisted of 16 sessions (8–12 weeks) with therapist-guided virtual-reality exercises, comprising reflections and challenges about the patients’ suspicious thoughts, safety behaviors, and harm expectancies. At this, the virtual social environments were individually designed for each patient, matching the idiosyncratic cues and paranoid fears of the individual patient. It goes without saying that the variations in the number, characteristics and responses of human avatars in VR would have not been controllable in real life exposure sessions. Overall, the findings of this recent RCT indicate that safety behaviors and modified social cognitions were mediators of treatment change and contributed to reductions in momentary paranoid ideation and anxiety (Pot-Kolder et al., 2018).

## Current Challenges and Implications for Future Research

However, to exploit the full potential of digitalized approaches to psychotherapy process research, it is essential to address several prevailing pitfalls and ethical considerations. These are, amongst others, fundamental data security, confidentiality, and emergency issues, as well as concerns in regard to certain unresolved research questions (Stoll et al., 2019). For example, a major confinement in IMIs is a comparatively high attrition rate and limited engagement of patients in these digital interventions, especially when they are unguided and transferred from controlled research settings into routine healthcare (Domhardt et al., 2019; Graham et al., 2019). Numerous research efforts are currently committed to find effective ways to increase the engagement—i.e., the frequency patients adopt and interact with IMIs (Graham et al., 2019)—such as user-centered design (Graham et al., 2019), product quality and therapeutic persuasiveness (Baumel and Kane, 2018), striving for higher completer rates and, as a consequence thereof, better treatment outcomes (Yardley et al., 2016). Likewise, several attempts and efforts are currently underway, in order to reach a better understanding of the attitudes of patients, therapists and stakeholders toward IMIs (Topooco et al., 2017; Apolinário-Hagen et al., 2018), as well as to establish legal and regulatory frameworks for the implementation of IMIs (Ebert et al., 2018), in order to pave the way for a broader dissemination of digital psychotherapeutic interventions in research and practice.

Aside these current challenges, there are conceptual and methodological confines that hampered the field of psychotherapy process research for decades. This holds true for divergent operationalizations of central constructs of psychotherapy research, which ancillary obstructed the long-lasting debate about the relative importance of common and specific factors (Mulder et al., 2017). For instance between “factors” and “components” that are part of the therapy (e.g., problem solving training), versus “mediators” and “mechanisms of change” that occur in the patient (e.g., application of newly acquired problem solving skills). Other examples of somewhat tenacious misconceptions in the literature are between “moderators” and “mediators” (Johansson and Høglend, 2007). Thus, next to the importance to stick to consistent operationalizations of existing constructs, it is also key to conceptualize certain unique features of digital interventions that might represent novel *digital common* or *digital specific factors*. Therewith we refer to factors that are *common* to all (e.g., technological design and delivery) or *specific* to certain digital health interventions (e.g., persuasive design, mobile sensing and ecological momentary interventions, and continuous automated feedback with smartphones or wearables)—but are not constituent of face-to-face psychotherapies. Future research must disclose, which of these digital factors are indeed active ingredients of technology-delivered interventions (or are merely facilitating or obstructive moderating variables for genuine therapeutic processes), and if they induce the same or separate working mechanisms when compared to conventional face-to-face psychotherapies. Albeit, these questions of comparative research are hardly to answer, as long as there are substantial differences between these two branches of research concerning recruitment strategies and sample characteristics (Torous and Firth, 2018). Another current confinement of digital approaches to psychotherapy research is their primary focus on interventions based on CBT-principles to this point (Andersson et al., 2019; Domhardt et al., 2020b). Hence, IMIs developed from other therapeutic backgrounds (such as psychodynamic, interpersonal or mindfulness-based approaches) are of value to expand the evidence base—therewith omitting an imbalance still observable in conventional psychotherapy research today (Leichsenring et al., 2018).

An additional major current concern lies in the light-minded interchange of correlation and causality with flawed conclusions on presumed psychological processes (Antes, 2016; Caliebe et al., 2019), as observed in some privately funded studies resorting to big data gathered by large tech companies. Hence, it is of utmost importance to comply with the traditional explanatory research sequence: hypothesize, model, and test (Anderson, 2008). Alongside the cautious contemplation of central notions of epistemology (i.e., verify vs. falsify; Carnap, 1928; Popper, 1959) and approaches to causal inference (Ohlsson and Kendler, 2019). Thereby, an attentive awareness of the differences between conventional and digitalized research methods in deriving knowledge from big data is of high relevance, as certain automated approaches lack testable hypotheses, conceptual frameworks or theoretical foundations (Kriston, 2020), as indispensable theoretical presuppositions for causal inferences (Wilkinson et al., 2020). As such, some methods relying on machine learning and artificial intelligence are not suitable to detect causal mechanisms in clinical settings, as they might impede transparency and replicability, which have to remain indispensable criteria for various decisions in healthcare. Hence, the consideration and advocacy of theory-driven explanatory research with falsifiable scientific models might be of particular relevance at the present time, so as to convey the scientific achievements and epistemological methodologies from decades of research efforts into an ever-increasing digitalized world, with the concomitant advancement of technologized psychological and medical research.

## Conclusion

Last but not least, in our view, the discussion about the opportunities and limitations of digitalized approaches to psychotherapy process research must not attend to technological and methodological aspects alone, but urgently needs to weigh the clinical and societal implications of their (non-)utilization hereafter. Accordingly, forthcoming research efforts ought to reveal, to which degree the innovations of digitalization will actually add *more light* on the mechanisms of change in psychotherapeutic interventions, and if we make the most out of technological opportunities to improve global mental health.

## Data Availability Statement

The original contributions presented in the study are included in the article/supplementary material, further inquiries can be directed to the corresponding author/s.

## Author Contributions

MD wrote the first draft of the manuscript. All authors have contributed to the further writing and have approved the final manuscript.

## Conflict of Interest

The authors declare that the research was conducted in the absence of any commercial or financial relationships that could be construed as a potential conflict of interest.
